# Supplementary material for: Integrative Physiological and Transcriptome Analysis Reveals the Mechanism for the Repair of Sub-Lethally Injured Escherichia coli O157:H7 Induced by High Hydrostatic Pressure
Source: Foods. 2022 Aug 8;11(15):2377. doi: 10.3390/foods11152377 (PMC9368309; doi:10.3390/foods11152377)
Supplement: Supplementary file 1 [file foods-11-02377-s001.zip › foods-1760481-supplementary.pdf]

Supplementary materials

Table S1. Information of primers for qRT-PCR.

Table S2. The sublethally injured (SI) cell contents (%) and SI rate (%) of different HHP conditions.

Table S3. Statistics of transcriptome analysis reads and assembly quality.

Table S4. Statistics of transcriptome analysis reads aligned to reference genome.

Table S5. The commonly up-regulated different expressed genes (DEGs).

Table S6. The commonly down-regulated DEGs.

Table S7. GO enrichment analysis of profile 1 and profile 2.

Table S8. KO enrichment analysis of profile 1 and profile 2.

Table S9. DEGs involved in pathways related to membrane.

Table S10. DEGs involved in pathways related to energy biosynthesis.

Table S11. DEGs involved in pathways related to genetic information transmission.

Table S12. DEGs involved in pathways related to other metabolisms.

Figure S1. The top 20 GO terms enriched in profile 1 and profile 2 (two significant clusters). Each fan-shaped region represented a GO term, the red square represented significance, while the purple square represented the amount of DEGs.

Table S1

Gene	Gene product	Primer sequence (5'-3')	Reference
16S rRNA	encoding 16S ribosome RNA	F: GGCTGAAAAGCTGCATTACC<br>R: CATCAGGCCGATGTTACCTT	[57]
accB	encoding acetyl CoA carboxylase	F: CTCAATCTAACGCAGCCG<br>R: TGACTTTCTGACCCACTTCG	This study
rpsS	encoding 30S ribosomal subunit protein S19	F: GAAAGCGGAGACAAGAAGC<br>R: CACCCAGTTTGTGACCAAC	This study
frdA	encoding fumarate reductase flavoprotein subunit	F: TCCTCAAACACACCCTCG<br>R: TCAGCCATTCGCCTTCTC	This study
atpG	encoding F0F1 ATP synthase subunit gamma	F: TGCTGCGTCGTTATGTCG<br>R: CGGTGAGTTCCTGAGTAATGC	This study
argG	encoding argininosuccinate synthetase	F: CACCATTGAGCAGTATCACG<br>R: TGGCTTGTAGGTCAGGTTC	This study
ansB	encoding L-asparaginase 2	F: GGCTAAAGCACTGGTAGATGC<br>R: TTTGGTTTGCGTCAGAGC	This study

Table S2

pressure (MPa)	time (min)	Intact cell content (%)	dead cell content (%)	SI cell content (%)	SI rate (%)
	1min	32.73±0.00^{b}	38.64±2.27^{e}	28.64±2.27^{b}	46.59±1.98^{c}
100 MPa	3min	71.18±4.68^{a}	18.23±3.45^{f}	10.59±1.23^{c}	13.04±2.06^{d}
	5min	42.17±3.99^{b}	23.60±8.22^{f}	34.23±4.23^{a}	44.73±0.73^{c}
	1min	22.22±1.34^{c}	46.05±0.44^{e}	31.73±0.91^{ab}	59.30±1.68^{c}
200 MPa	3min	13.46±10.32^{c}	74.12±12.72^{cd}	12.42±2.40^{c}	57.27±18.86^{c}
	5min	17.24±1.27^{c}	67.54±0.88^{d}	15.22±2.15^{c}	46.75±5.36^{c}
	1min	2.08±0.10^{d}	82.89±0.44^{bc}	15.02±0.54^{c}	87.80±0.90^{ab}
300 MPa	3min	0.98±0.05^{d}	94.94±0.13^{ab}	4.08±0.18^{d}	80.61±1.39^{b}
	5min	0.27±0.06^{d}	97.78±0.31^{a}	1.95±0.25^{d}	88.09±1.16^{ab}
	1min	0.13±0.03^{d}	98.08±0.17^{a}	1.80±0.14^{d}	93.48±1.10^{ab}
400 MPa	3min	0.01±0.00^{d}	99.68±0.17^{a}	0.31±0.17^{d}	98.32±0.04^{a}
	5min	0.00±0.00^{d}	99.74±0.02^{a}	0.25±0.02^{d}	99.44±0.02^{a}
	1min	0.00±0.00^{d}	99.95±0.00^{a}	0.05±0.00^{d}	99.87±0.00^{a}
500 MPa	3min	0.00±0.00^{d}	100.00±0.00^{a}	0.00±0.00^{d}	99.99±0.00^{a}
	5min	0.00±0.00^{d}	100.00±0.00^{a}	0.00±0.00^{d}	100.00±0.00^{a}

Different letters indicate statistically significance among groups treated at the same pressure and different times ($p \leq 0.05$, Duncan test).

Table S3

Sample	Before Filter			After Filter		
	Raw Data(bp)	Q20(%)	Q30(%)	Clean Data(bp)	Q20(%)	Q30(%)
UT-1	1067811900	1045934263 (97.95%)	1003749164 (94.0%)	1003731547	984188338 (98.05%)	945515674 (94.2%)
UT-2	1039411800	1017320445 (97.87%)	975330581 (93.83%)	976476147	956941579 (98.0%)	918448032 (94.06%)
UT-3	1051873800	1031755554 (98.09%)	991946968 (94.3%)	988214973	970248312 (98.18%)	933805264 (94.49%)
HHP-1	1093588200	1072806598 (98.1%)	1030692123 (94.25%)	1029524305	1011408633 (98.24%)	972932788 (94.5%)
HHP-2	921642600	906703989 (98.38%)	874062411 (94.84%)	866564174	853461933 (98.49%)	823907007 (95.08%)
HHP-3	890157000	875556300 (98.36%)	843864120 (94.8%)	839768921	826797263 (98.46%)	797781539 (95.0%)
Repair-1	922627500	907701195 (98.38%)	875045092 (94.84%)	861150652	848166939 (98.49%)	818899924 (95.09%)
Repair-2	948244200	931824631 (98.27%)	897006969 (94.6%)	888582204	874216363 (98.38%)	842791206 (94.85%)
Repair-3	1022214600	1004279477 (98.25%)	966484825 (94.55%)	960481371	944589446 (98.35%)	910197783 (94.76%)

Table S4

**Sample**	**Total Reads**	**Unmapped Reads**	**Unique Mapped Reads**	**Multiple Mapped reads**	**Mapping Ratio**
UT-1	6367292	517692(8.13%)	5774713(90.69%)	74887(1.18%)	91.87%
UT-2	6242734	492577(7.89%)	5673474(90.88%)	76683(1.23%)	92.11%
UT-3	6374502	512297(8.04%)	5785978(90.77%)	76227(1.20%)	91.96%
HHP-1	6063864	907306(14.96%)	5049276(83.27%)	107282(1.77%)	85.04%
HHP-2	5470744	543258(9.93%)	4832476(88.33%)	95010(1.74%)	90.07%
HHP-3	5290698	518270(9.80%)	4682595(88.51%)	89833(1.70%)	90.20%
Repair-1	5784618	810608(14.01%)	4709900(81.42%)	264110(4.57%)	85.99%
Repair-2	5971414	836346(14.01%)	4862554(81.43%)	272514(4.56%)	85.99%
Repair-3	6359572	905020(14.23%)	5163245(81.19%)	291307(4.58%)	85.77%

Table S5

Gene id	Gene name	UT-vs-HHP		HHP-vs-Repair		UT-vs-Repair	
		log_2FC	fdr	log_2FC	fdr	log_2FC	fdr
b0614	*citX*	2.33	3.54×10^{-17}	1.04	1.81×10^{-9}	3.37	2.30×10^{-117}
b0615	*citF*	1.52	4.97×10^{-15}	1.73	3.40×10^{-45}	3.25	1.75×10^{-302}
b0693	*speF*	2.41	7.51×10^{-50}	1.74	7.17×10^{-62}	4.15	0
b0903	*pflB*	1.06	5.01×10^{-151}	2.07	0	3.13	0
b1779	*gapA*	1.27	1.56×10^{-153}	1.56	0	2.83	0
b2168	*fruK*	5.93	0	1.27	3.61×10^{-124}	7.20	0
b2169	*fruB*	4.58	9.05×10^{-263}	2.96	0	7.54	0
b2719	*hycG*	1.53	7.07×10^{-4}	1.85	6.61×10^{-14}	3.38	1.82×10^{-76}
b2720	*hycF*	1.13	1.70×10^{-2}	2.25	6.89×10^{-19}	3.38	1.02×10^{-83}
b2721	*hycE*	2.18	1.64×10^{-9}	1.85	3.39×10^{-27}	4.03	7.40×10^{-255}
b2722	*hycD*	1.93	3.89×10^{-4}	1.55	7.30×10^{-8}	3.48	3.03×10^{-52}
b2723	*hycC*	1.54	7.89×10^{-10}	1.32	4.16×10^{-16}	2.86	7.17×10^{-121}
b2724	*hycB*	1.74	3.89×10^{-2}	1.40	7.82×10^{-3}	3.14	2.34×10^{-16}
b2943	*galP*	1.29	1.38×10^{-5}	1.47	2.78×10^{-13}	2.76	6.78×10^{-75}
b3366	*nirD*	1.25	3.89×10^{-2}	2.15	1.66×10^{-11}	3.41	1.11×10^{-51}
b4484	*cpxP*	3.33	7.76×10^{-43}	1.40	1.37×10^{-25}	4.73	6.43×10^{-300}

Table S6

Gene id	Gene name	UT-vs-HHP		HHP-vs-Repair		UT-vs-Repair	
		$\log_2$FC	fdr	$\log_2$FC	fdr	$\log_2$FC	fdr
b2255	*arnA*	-2.58	2.89×10^{-125}	-1.96	1.27×10^{-22}	-4.53	0
b2253	*arnB*	-2.36	2.44×10^{-118}	-1.87	2.65×10^{-27}	-4.23	0
b2254	*arnC*	-2.81	1.41×10^{-74}	-2.43	5.88×10^{-13}	-5.24	0
b2257	*arnT*	-1.20	4.19×10^{-4}	-1.98	3.93×10^{-3}	-3.19	3.54×10^{-31}
b0112	*aroP*	-1.52	3.18×10^{-23}	-1.16	5.76×10^{-6}	-2.68	2.65×10^{-139}
b1747	*astA*	-1.94	1.83×10^{-12}	-3.85	4.27×10^{-8}	-5.79	2.77×10^{-85}
b1745	*astB*	-1.43	1.15×10^{-5}	-3.16	1.60×10^{-5}	-4.58	6.81×10^{-55}
b1748	*astC*	-1.47	8.18×10^{-5}	-2.42	9.83×10^{-4}	-3.89	3.01×10^{-35}
b1746	*astD*	-1.44	8.11×10^{-7}	-3.45	2.49×10^{-7}	-4.89	1.60×10^{-65}
b1744	*astE*	-1.55	8.34×10^{-3}	-2.55	3.37×10^{-2}	-4.10	7.74×10^{-19}
b4208	*cycA*	-1.45	6.69×10^{-56}	-1.25	6.04×10^{-21}	-2.69	0
b0430	*cyoC*	-1.12	9.35×10^{-33}	-1.89	5.72×10^{-42}	-3.01	0
b3845	*fadA*	-1.79	7.43×10^{-134}	-5.36	1.19×10^{-205}	-7.14	0
b3846	*fadB*	-2.05	2.21×10^{-296}	-1.89	4.68×10^{-98}	-3.94	0
b0221	*fadE*	-1.12	1.95×10^{-160}	-3.45	0	-4.57	0
b2341	*fadJ*	-2.28	3.58×10^{-250}	-1.90	5.64×10^{-60}	-4.18	0
b2344	*fadL*	-1.82	2.72×10^{-93}	-2.09	1.48×10^{-45}	-3.91	0
b0654	*gltJ*	-2.49	2.30×10^{-23}	-2.39	4.44×10^{-4}	-4.88	2.98×10^{-121}
b0653	*gltK*	-2.54	3.69×10^{-39}	-2.44	3.59×10^{-7}	-4.98	2.28×10^{-191}
b0652	*gltL*	-1.47	8.68×10^{-15}	-3.42	8.65×10^{-19}	-4.89	8.16×10^{-132}
b0071	*leuD*	-1.36	1.59×10^{-3}	-2.15	2.21×10^{-2}	-3.51	4.84×10^{-28}
b0346	*mhpR*	-2.55	1.92×10^{-26}	-1.70	1.01×10^{-3}	-4.25	2.69×10^{-112}
b1988	*nac*	-2.05	2.88×10^{-7}	-1.76	2.59×10^{-2}	-3.81	8.91×10^{-36}
b3225	*nanA*	-1.13	1.09×10^{-15}	-2.63	1.77×10^{-41}	-3.76	5.57×10^{-165}
b3223	*nanE*	-1.57	2.15×10^{-6}	-2.86	5.76×10^{-4}	-4.43	3.22×10^{-54}
b3224	*nanT*	-1.98	4.75×10^{-22}	-3.51	2.63×10^{-10}	-5.49	5.20×10^{-144}
b2616	*recN*	-1.98	8.42×10^{-34}	-1.29	7.37×10^{-5}	-3.27	8.84×10^{-189}
b1012	*rutA*	-1.46	6.97×10^{-7}	-5.02	8.30×10^{-11}	-6.49	2.03×10^{-71}
b1009	*rutD*	-1.65	1.70×10^{-2}	-4.54	2.50×10^{-2}	-6.19	7.05×10^{-18}
b2340	*sixA*	-2.80	1.52×10^{-40}	-1.15	4.04×10^{-3}	-3.94	5.54×10^{-140}
b4062	*soxS*	-2.49	1.05×10^{-210}	-1.78	2.04×10^{-43}	-4.27	0
b1783	*yeaG*	-1.75	1.10×10^{-162}	-3.84	1.50×10^{-191}	-5.59	0
b1784	*yeaH*	-3.92	1.86×10^{-159}	-1.80	1.26×10^{-7}	-5.72	0
b2292	*yfbS*	-1.29	2.49×10^{-24}	-1.45	3.38×10^{-15}	-2.74	5.86×10^{-172}
b2398	*yfeC*	-1.82	1.46×10^{-25}	-1.28	1.35×10^{-5}	-3.10	7.59×10^{-125}

Table S7

Ontology	GO ID	Description	profile1 (157)	All (1280)	p-value	p-adjust
profile 1						
Cellular Component	GO:0016020	membrane	92 (58.6%)	586 (45.78%)	0.000402	0.010625
	GO:0044425	membrane part	79 (50.32%)	485 (37.89%)	0.000483	0.010625
	GO:0031224	intrinsic component of membrane	77 (49.04%)	477 (37.27%)	0.000865	0.012692
Biological Process	GO:0006551	leucine metabolic process	4 (1.45%)	5 (0.21%)	0.000833	0.277322
	GO:0005976	polysaccharide metabolic process	10 (3.62%)	29 (1.23%)	0.001067	0.277322
	GO:0044264	cellular polysaccharide metabolic process	8 (2.9%)	25 (1.06%)	0.00573	0.322242
	GO:0015893	drug transport	3 (1.09%)	4 (0.17%)	0.005786	0.322242
	GO:0034762	regulation of transmembrane transport	3 (1.09%)	4 (0.17%)	0.005786	0.322242
	GO:0034765	regulation of ion transmembrane transport	3 (1.09%)	4 (0.17%)	0.005786	0.322242
	GO:0042493	response to drug	3 (1.09%)	4 (0.17%)	0.005786	0.322242
	GO:0071941	nitrogen cycle metabolic process	3 (1.09%)	4 (0.17%)	0.005786	0.322242
	GO:0000910	cytokinesis	6 (2.17%)	17 (0.72%)	0.009785	0.322242
	GO:0007049	cell cycle	6 (2.17%)	17 (0.72%)	0.009785	0.322242
	GO:0022402	cell cycle process	6 (2.17%)	17 (0.72%)	0.009785	0.322242
	GO:0051301	cell division	6 (2.17%)	17 (0.72%)	0.009785	0.322242
	GO:0006073	cellular glucan metabolic process	5 (1.81%)	13 (0.55%)	0.012273	0.322242
	GO:0044042	glucan metabolic process	5 (1.81%)	13 (0.55%)	0.012273	0.322242
	GO:0051181	cofactor transport	3 (1.09%)	5 (0.21%)	0.013217	0.322242
	GO:0006101	citrate metabolic process	6 (2.17%)	18 (0.76%)	0.013268	0.322242
	GO:0009081	branched-chain amino acid metabolic process	6 (2.17%)	18 (0.76%)	0.013268	0.322242
profile 2						
Biological Process	GO:0044281	small molecule metabolic process	160 (30.71%)	515 (21.82%)	0	0.000029
	GO:1901564	organonitrogen compound metabolic process	127 (24.38%)	391 (16.57%)	0	0.000037
	GO:1901566	organonitrogen compound biosynthetic process	68 (13.05%)	180 (7.63%)	0	0.000103

	GO:0044710	single-organism metabolic process	240 (46.07%)	870 (36.86%)	0.000001	0.000112
	GO:0044271	cellular nitrogen compound biosynthetic process	66 (12.67%)	176 (7.46%)	0.000001	0.000115
	GO:0008152	metabolic process	436 (83.69%)	1793 (75.97%)	0.000001	0.000115
Molecular Function	GO:0005198	structural molecule activity	36 (7.06%)	56 (2.48%)	0	0
	GO:0003723	RNA binding	40 (7.84%)	73 (3.24%)	0	0
	GO:0005488	binding	335 (65.69%)	1233 (54.68%)	0	0.000001
	GO:0036094	small molecule binding	158 (30.98%)	497 (22.04%)	0	0.000004
	GO:1901363	heterocyclic compound binding	217 (42.55%)	756 (33.53%)	0.000001	0.000053
	GO:0097159	organic cyclic compound binding	219 (42.94%)	782 (34.68%)	0.000007	0.000354
Cellular Component	GO:0030529	intracellular ribonucleoprotein complex	38 (12.42%)	60 (4.69%)	0	0
	GO:1990904	ribonucleoprotein complex	38 (12.42%)	60 (4.69%)	0	0
	GO:0005622	intracellular	184 (60.13%)	570 (44.53%)	0	0
	GO:0044424	intracellular part	184 (60.13%)	570 (44.53%)	0	0
	GO:0005623	cell	217 (70.92%)	730 (57.03%)	0	0
	GO:0044464	cell part	217 (70.92%)	730 (57.03%)	0	0
	GO:0032991	macromolecular complex	56 (18.3%)	146 (11.41%)	0.000024	0.000176
	GO:0044446	intracellular organelle part	15 (4.9%)	24 (1.88%)	0.000054	0.00035

Table S8

**Pathway**	***P*-value**	**Q-value**	**Pathway ID**
profile 1			
C5-Branched dibasic acid metabolism	0.00026	0.0224	ko00660
Cell cycle - Caulobacter	0.00439	0.124	ko04112
Valine, leucine and isoleucine biosynthesis	0.00556	0.124	ko00290
ABC transporters	0.00601	0.124	ko02010
Citrate cycle (TCA cycle)	0.0072	0.124	ko00020
Quorum sensing	0.0125	0.159	ko02024
Peptidoglycan biosynthesis	0.013	0.159	ko00550
2-Oxocarboxylic acid metabolism	0.0203	0.218	ko01210
Carbon fixation pathways in prokaryotes	0.025	0.239	ko00720
profile 2			
Ribosome	0	0.0000000978	ko03010
Alanine, aspartate and glutamate metabolism	0.00135	0.0718	ko00250
Aminoacyl-tRNA biosynthesis	0.00454	0.16	ko00970
Terpenoid backbone biosynthesis	0.00707	0.187	ko00900
Arginine biosynthesis	0.0241	0.444	ko00220
Biosynthesis of secondary metabolites	0.0281	0.444	ko01110
Fatty acid biosynthesis	0.0293	0.444	ko00061
Oxidative phosphorylation	0.0340	0.450	ko00190

Table S9

Gene ID	Gene name	UT-vs-HHP		HHP-vs-Repair		Desceiption
		**log_2FC**	**p-value**	**log_2FC**	**p-value**	
Peptidoglycan biosynthesis						
b3189	*murA*	-1.07	3.98×10^{-4}	1.73	6.26×10^{-12}	UDP-N-acetylglucosamine 1-carboxyvinyltransferase
b3972	*murB*	-1.05	9.23×10^{-3}	1.05	7.32×10^{-3}	UDP-N-acetylenolpyruvoylglucosamine reductase
b0091	*murC*	-2.81	2.04×10^{-39}	2.13	1.49×10^{-19}	UDP-N-acetylmuramate--L-alanine ligase
b0088	*murD*	-1.64	8.96×10^{-16}	0.89	9.63×10^{-5}	UDP-N-acetylmuramoylalanine--D-glutamate ligase
b0839	*dacC*	-1.31	8.55×10^{-5}	0.29	5.12×10^{-1}	D-alanyl-D-alanine carboxypeptidase
b0092	*ddlB*	-1.51	2.27×10^{-19}	0.29	1.63×10^{-1}	D-alanine--D-alanine ligase B
b0084	*ftsI*	-1.74	4.06×10^{-24}	0.04	9.14×10^{-1}	cell division protein FtsL
b0087	*mraY*	-2.53	4.06×10^{-32}	1.16	7.21×10^{-5}	UDP-N-acetylmuramoylalanyl-D-glutamyl-2, 6-diaminopimelate--D-alanalanyl ligase
b0085	*murE*	-1.83	4.84×10^{-20}	0.82	5.48×10^{-4}	peptidoglycan synthetase FtsI
b0086	*murF*	-2.12	8.83×10^{-30}	1.36	4.45×10^{-10}	D-alanyl-D-alanine-adding enzyme
b0090	*murG*	-2.20	6.15×10^{-19}	0.65	6.17×10^{-2}	N-acetylglucosaminyl transferase
b2430	*yfeW*	-1.35	8.00×10^{-9}	0.67	1.26×10^{-2}	penicillin binding protein 4B
Fatty acid biosynthesis						
b0185	*accA*	-1.61	6.88×10^{-7}	2.11	1.11×10^{-12}	acetyl-CoA carboxylase carboxyl transferase alpha subunit
b3255	*accB*	-3.45	2.63×10^{-57}	2.14	1.55×10^{-14}	acetyl CoA carboxylase
b3256	*accC*	-2.54	6.06×10^{-64}	1.96	7.03×10^{-38}	Biotin carboxylase
b1091	*fabH*	-2.19	3.12×10^{-26}	1.29	5.27×10^{-8}	3-oxoacyl-[acyl-carrier-protein] synthase III
b1092	*fabD*	-1.71	1.17×10^{-22}	1.44	2.18×10^{-15}	[acyl-carrier-protein] S-malonyltransferase
b1093	*fabG*	-1.44	2.13×10^{-35}	0.91	1.78×10^{-12}	3-oxoacyl-[acyl-carrier-protein] reductase FabG
b1095	*fabF*	-2.32	2.27×10^{-72}	1.17	1.47×10^{-16}	3-oxoacyl-[acyl-carrier-protein] synthase II
b2323	*fabB*	0.16	2.04×10^{-1}	1.43	4.80×10^{-59}	beta-ketoacyl-[acyl carrier protein] synthase I
b1805	*fadD*	-3.19	3.42×10^{-143}	-0.62	6.70×10^{-3}	long-chain-fatty-acid--CoA ligase FadD

Table S10

Gene ID	Gene name	UT-vs-HHP		HHP-vs-Repair		Desceiption
		log_2FC	p-value	log_2FC	p-value	
b0428	*cyoE*	-0.56	1.08×10^{-23}	-3.07	2.22×10^{-272}	cytochrome o ubiquinol oxidase C subunit
b0429	*cyoD*	-0.93	2.43×10^{-10}	-3.13	5.01×10^{-36}	cytochrome o ubiquinol oxidase subunit IV
b0430	*cyoC*	-1.12	8.84×10^{-34}	-1.89	3.29×10^{-43}	cytochrome bo3 ubiquinol oxidase subunit 3
b0722	*sdhD*	-1.37	5.23×10^{-24}	-0.31	1.11×10^{-1}	succinate:quinone oxidoreductase, membrane protein SdhD
b0723	*sdhA*	-1.86	6.36×10^{-162}	0.22	6.20×10^{-3}	succinate dehydrogenase flavoprotein subunit
b0724	*sdhB*	-1.70	4.96×10^{-119}	-0.45	8.10×10^{-6}	succinate:quinone oxidoreductase, iron-sulfur cluster binding protein
b0978	*appC*	-1.83	4.57×10^{-20}	4.19	5.97×10^{-202}	Cytochrome d ubiquinol oxidase subunit I
b0979	*appB*	-2.11	5.44×10^{-21}	4.23	5.24×10^{-156}	cytochrome bd-II oxidase subunit 2
b1109	*ndh*	-0.40	2.20×10^{-1}	3.60	3.08×10^{-103}	NADH dehydrogenase
b2280	*nuoJ*	-1.34	4.21×10^{-9}	1.04	2.36×10^{-5}	NADH-quinone oxidoreductase subunit J
b2281	*nuoI*	-1.38	1.97×10^{-12}	0.59	1.48×10^{-2}	NADH:ubiquinone oxidoreductase, chain I protein
b2282	*nuoH*	-1.16	5.92×10^{-17}	0.60	1.88×10^{-4}	NADH-quinone oxidoreductase subunit H
b2283	*nuoG*	-1.75	2.98×10^{-43}	1.40	2.50×10^{-34}	NADH:ubiquinone oxidoreductase, chain G
b2284	*nuoF*	-1.83	4.03×10^{-13}	1.20	3.40×10^{-7}	NADH:quinone oxidoreductase subunit F
b2285	*nuoE*	-1.75	4.20×10^{-4}	1.24	2.18×10^{-2}	NADH-quinone oxidoreductase subunit NuoE
b2286	*nuoC*	-2.65	1.48×10^{-39}	2.25	2.96×10^{-26}	RecName: Full=NADH-quinone oxidoreductase subunit C/D; AltName: Full=NADH dehydrogenase I subunit C/D; AltName: Full=NDH-1 subunit C/D
b2287	*nuoB*	-1.90	3.59×10^{-19}	1.44	2.60×10^{-10}	NADH-quinone oxidoreductase subunit NuoB
b2288	*nuoA*	-1.69	2.96×10^{-10}	0.22	5.85×10^{-1}	NADH-quinone oxidoreductase subunit A
b2501	*ppk*	-2.02	7.25×10^{-36}	1.32	1.74×10^{-13}	polyphosphate kinase
b3731	*atpC*	-1.22	1.72×10^{-13}	0.94	2.54×10^{-8}	ATP synthase F1 complex subunit epsilon
b3732	*atpD*	-2.08	3.38×10^{-77}	1.80	5.50×10^{-52}	F0F1 ATP synthase subunit beta
b3733	*atpG*	-2.38	3.21×10^{-50}	2.02	1.09×10^{-31}	F0F1 ATP synthase subunit gamma
b3734	*atpA*	-1.29	1.43×10^{-30}	1.10	3.04×10^{-22}	F0F1 ATP synthase subunit alpha
b3735	*atpH*	-1.66	3.51×10^{-9}	0.64	6.39×10^{-2}	F0F1 ATP synthase subunit delta
b4151	*frdD*	-1.24	1.47×10^{-17}	1.11	9.41×10^{-13}	fumarate reductase membrane anchor subunit
b4152	*frdC*	-1.58	1.15×10^{-38}	1.23	3.79×10^{-21}	fumarate reductase subunit FrdC
b4153	*frdB*	-2.75	1.30×10^{-124}	2.74	2.74×10^{-96}	frdB
b4154	*frdA*	-3.22	5.40×10^{-268}	3.02	6.83×10^{-206}	fumarate reductase flavoprotein subunit
b4226	*ppa*	-0.77	1.46×10^{-2}	1.37	4.69×10^{-7}	inorganic diphosphatase, partial

Table S11

Gene ID	Gene name	UT-vs-HHP		HHP-vs-Repair		Desceiption
		log_2FC	p-value	log_2FC	p-value	
Aminoacyl-tRNA biosynthesis						
b0026	*ileS*	-0.97	1.85×10^{-11}	1.44	2.87×10^{-25}	isoleucine--tRNA ligase
b0194	*proS*	-1.16	2.96×10^{-6}	2.24	8.09×10^{-26}	proline--tRNA ligase
b0526	*cysS*	-0.97	5.04×10^{-2}	2.06	2.16×10^{-7}	cysteine--tRNA ligase
b0642	*leuS*	-1.63	7.54×10^{-24}	2.27	9.56×10^{-63}	Leucyl-tRNA synthetase
b0680	*glnS*	-0.85	5.06×10^{-4}	1.88	8.99×10^{-20}	glutamine--tRNA ligase
b0893	*serS*	-2.18	6.78×10^{-99}	1.33	4.24×10^{-45}	serine--tRNA ligase, partial
b0930	*asnS*	-1.76	1.27×10^{-24}	0.86	1.45×10^{-5}	asparagine--tRNA ligase
b1637	*tyrS*	-0.44	4.15×10^{-3}	1.43	3.18×10^{-27}	tyrosine--tRNA ligase
b1714	*pheS*	-0.92	2.09×10^{-2}	2.57	8.80×10^{-21}	phenylalanine--tRNA ligase, alpha subunit
b1719	*thrS*	-1.64	5.59×10^{-111}	1.07	9.35×10^{-41}	threonine--tRNA ligase
b1866	*aspS*	-1.47	3.07×10^{-26}	1.76	3.22×10^{-33}	aspartate--tRNA ligase
b1876	*argS*	-1.29	3.75×10^{-4}	2.20	3.65×10^{-13}	arginine--tRNA ligase
b2114	*metG*	-0.51	1.05×10^{-2}	1.21	2.69×10^{-11}	methionine--tRNA ligase
b2400	*gltX*	-1.22	2.78×10^{-8}	2.40	5.73×10^{-41}	glutamate--tRNA ligase
b2514	*hisS*	-1.03	9.66×10^{-10}	1.06	6.92×10^{-11}	histidyl tRNA synthetase
b2697	*alaS*	-1.15	3.07×10^{-11}	2.47	1.80×10^{-68}	alanine--tRNA ligase/DNA-binding transcriptional repressor
b2890	*lysS*	-1.37	1.38×10^{-5}	2.24	1.30×10^{-26}	lysine--tRNA ligase, constitutive
b3288	*fmt*	-1.17	1.26×10^{-25}	0.52	6.60×10^{-6}	10-formyltetrahydrofolate:L-methionyl-tRNA(fMet) N-formyltransferase
b3384	*trpS*	-1.53	1.37×10^{-8}	1.90	4.60×10^{-14}	tryptophan--tRNA ligase
b3559	*glyS*	-0.87	3.36×10^{-5}	1.29	1.32×10^{-12}	glycine--tRNA ligase subunit beta
b3591	*selA*	-2.39	6.77×10^{-9}	1.69	1.93×10^{-4}	L-seryl-tRNA(Sec) selenium transferase
b4129	*lysU*	0.10	2.98×10^{-1}	2.31	2.41×10^{-225}	lysine--tRNA ligase
b4258	*valS*	-0.52	5.15×10^{-4}	1.23	4.66×10^{-20}	valine--tRNA ligase
Ribosome						
b3312	*rpmC*	-2.00	5.47×10^{-33}	1.62	1.56×10^{-19}	50S ribosomal subunit protein L29
b3313	*rplP*	-2.35	5.86×10^{-96}	1.98	5.61×10^{-52}	50S ribosomal subunit protein L16
b3314	*rpsC*	-2.03	1.01×10^{-97}	1.79	6.39×10^{-70}	30S ribosomal subunit protein S3
b3315	*rplV*	-2.47	8.67×10^{-33}	2.46	4.57×10^{-32}	50S ribosomal subunit protein L22
b3316	*rpsS*	-2.51	2.66×10^{-43}	2.61	4.60×10^{-47}	30S ribosomal subunit protein S19
b3317	*rplB*	-1.56	4.08×10^{-40}	2.09	1.15×10^{-85}	50S ribosomal protein L2
b3318	*rplW*	-1.53	1.98×10^{-15}	1.81	1.03×10^{-22}	50S ribosomal subunit protein L23
b3319	*rplD*	-1.83	3.75×10^{-34}	2.22	2.02×10^{-52}	50S ribosomal subunit protein L4
b3320	*rplC*	-1.41	2.03×10^{-24}	1.44	2.24×10^{-26}	50S ribosomal subunit protein L3
b3321	*rpsJ*	-1.31	5.13×10^{-11}	1.44	7.32×10^{-14}	30S ribosomal subunit protein S10
b3341	*rpsG*	-1.23	1.13×10^{-14}	1.32	6.59×10^{-17}	30S ribosomal subunit protein S7
b3342	*rpsL*	-0.65	1.83×10^{-3}	1.07	2.80×10^{-08}	30S ribosomal protein S12
b3301	*rplO*	-1.96	2.21×10^{-102}	1.35	2.03×10^{-36}	50S ribosomal subunit protein L15
b3302	*rpmD*	-1.68	3.44×10^{-6}	1.40	4.75×10^{-5}	50S ribosomal subunit protein L30
b3303	*rpsE*	-1.76	2.87×10^{-82}	1.45	1.63×10^{-51}	30S ribosomal subunit protein S5
b3304	*rplR*	-2.01	1.03×10^{-42}	1.90	6.94×10^{-38}	50S ribosomal subunit protein L18

b3305	*rplF*	-1.24	9.13×10^{-26}	1.60	7.20 × 10-41	50S ribosomal subunit protein L6
b3306	*rpsH*	-2.03	2.69×10^{-23}	2.12	2.33×10^{-25}	30S ribosomal subunit protein S8
b3307	*rpsN*	-1.76	3.25×10^{-26}	2.18	1.65×10^{-52}	30S ribosomal subunit protein S14
b3308	*rplE*	-0.53	2.48×10^{-7}	1.07	2.42×10^{-29}	50S ribosomal subunit protein L5
b3309	*rplX*	-0.94	4.41×10^{-4}	1.16	3.88×10^{-6}	50S ribosomal subunit protein L24
b3311	*rpsQ*	-1.43	8.07×10^{-40}	1.19	7.02×10^{-26}	30S ribosomal protein S17
b3296	*rpsD*	-2.42	2.49×10^{-77}	2.17	1.50×10^{-57}	30S ribosomal subunit protein S4
b3297	*rpsK*	-1.19	2.96×10^{-24}	0.87	8.14×10^{-12}	30S ribosomal subunit protein S11
b3298	*rpsM*	-1.59	2.40×10^{-26}	1.45	6.06×10^{-21}	30S ribosomal subunit protein S13
b3230	*rpsI*	-1.34	2.32×10^{-20}	1.11	4.12×10^{-15}	rpsI protein
b3231	*rplM*	-1.43	1.04×10^{-13}	1.69	1.93×10^{-22}	50S ribosomal protein L13
b3294	*rplQ*	-1.89	8.49×10^{-38}	1.36	5.34×10^{-17}	50S ribosomal subunit protein L17
b0169	*rpsB*	-0.76	3.74×10^{-9}	1.77	1.12×10^{-54}	30S ribosomal subunit protein S2
b1717	*rpmI*	-0.78	2.05×10^{-5}	1.18	5.37×10^{-12}	50S ribosomal protein L35
b0911	*rpsA*	-0.94	2.59×10^{-53}	1.36	8.00×10^{-122}	30S ribosomal subunit protein S1
b2185	*rplY*	-2.75	1.79×10^{-26}	2.19	2.10×10^{-16}	ribosomal L25p family protein
b2606	*rplS*	-3.57	1.67×10^{-36}	1.79	2.19×10^{-5}	50S ribosomal subunit protein L19
b2609	*rpsP*	-1.55	2.78×10^{-3}	0.53	3.52×10^{-1}	30S ribosomal protein S16
b3065	*rpsU*	2.55	7.39×10^{-4}	-2.09	2.21×10^{-3}	30S ribosomal protein S21
b3165	*rpsO*	-2.04	7.21×10^{-17}	0.68	1.51×10^{-2}	30S ribosomal subunit protein S15
b3983	*rplK*	-0.49	1.51×10^{-2}	1.46	1.98×10^{-17}	50S ribosomal subunit protein L11
b3984	*rplA*	-0.39	4.10×10^{-3}	1.98	8.75×10^{-74}	50S ribosomal subunit protein L1
b4200	*rpsF*	-2.05	5.51×10^{-43}	1.92	2.22×10^{-36}	30S ribosomal subunit protein S6
b4202	*rpsR*	-1.41	1.19×10^{-4}	0.62	2.28×10^{-1}	30S ribosomal subunit protein S18
b4203	*rplI*	-2.61	1.41×10^{-50}	2.13	6.75×10^{-29}	50S ribosomal subunit protein L9
b0296	*rpmE2-1*	-15.66	3.48×10^{-4}	16.20	8.48×10^{-6}	50S ribosomal protein L31 type B
b3936	*rpmE*	-1.59	1.28×10^{-6}	1.01	3.62×10^{-3}	50S ribosomal subunit protein L31
b3637	*rpmB*	-1.17	3.05×10^{-4}	0.93	2.07×10^{-2}	50S ribosomal subunit protein L28

Table S12

Gene ID	Gene name	UT-vs-HHP		HHP-vs-Repair		Desceiption
		log_2FC	p-value	log_2FC	p-value	
Valine, leucine and isoleucine biosynthesis						
b0071	*leuD*	-1.36	5.54×10^{-4}	-2.15	8.14×10^{-3}	3-isopropylmalate dehydratase subunit LeuD
b0072	*leuC*	-2.55	7.85×10^{-13}	0.42	4.72×10^{-1}	3-isopropylmalate dehydratase subunit LeuC
b0073	*leuB*	-1.96	3.46×10^{-5}	0.08	1.00	2-isopropylmalate synthase
b0074	*leuA*	-1.84	4.27×10^{-7}	0.41	4.59×10^{-1}	2-isopropylmalate synthase
b0077	*ilvI*	-2.54	8.50×10^{-7}	1.61	2.20×10^{-2}	acetolactate synthase, large subunit, biosynthetic type
b0078	*ilvH*	-3.70	3.60×10^{-7}	1.81	8.37×10^{-2}	acetolactate synthase 3 regulatory subunit
b2290	*alaA*	-2.73	2.83×10^{-73}	1.74	1.01×10^{-23}	glutamate--pyruvate aminotransferase AlaA
b3117	*tdcB*	-6.54	1.01×10^{-62}	4.57	1.62×10^{-15}	bifunctional threonine ammonia-lyase/L-serine ammonia-lyase TdcB
b3572	*avtA*	-1.78	1.42×10^{-7}	1.82	8.72×10^{-8}	valine--pyruvate transaminase
b3670	*ilvN*	0.45	3.19×10^{-1}	-2.21	1.17×10^{-5}	acetolactate synthase 1 small subunit
b3671	*ilvB*	2.11	2.87×10^{-37}	-2.09	4.47×10^{-36}	acetohydroxy acid synthase I subunit IlvB
b3769	*ilvM*	-2.60	2.97×10^{-4}	0.14	1.00	acetolactate synthase 2 small subunit
b3770	*ilvE*	-1.13	2.05×10^{-10}	0.23	2.68×10^{-1}	Branched-chain amino acid aminotransferase
b3771	*ilvD*	-1.03	5.86×10^{-5}	0.59	2.04×10^{-2}	dihydroxy-acid dehydratase
b3774	*ilvC*	-1.38	5.33×10^{-7}	1.25	6.37×10^{-6}	ketol-acid reductoisomerase

Profile 1

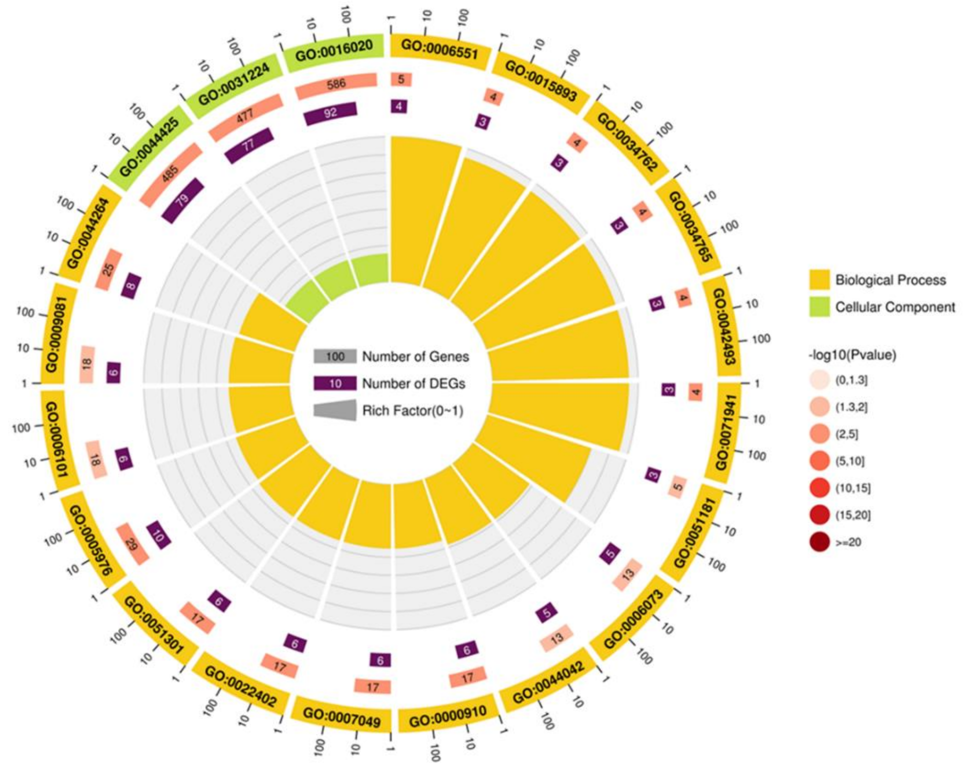

Profile 2

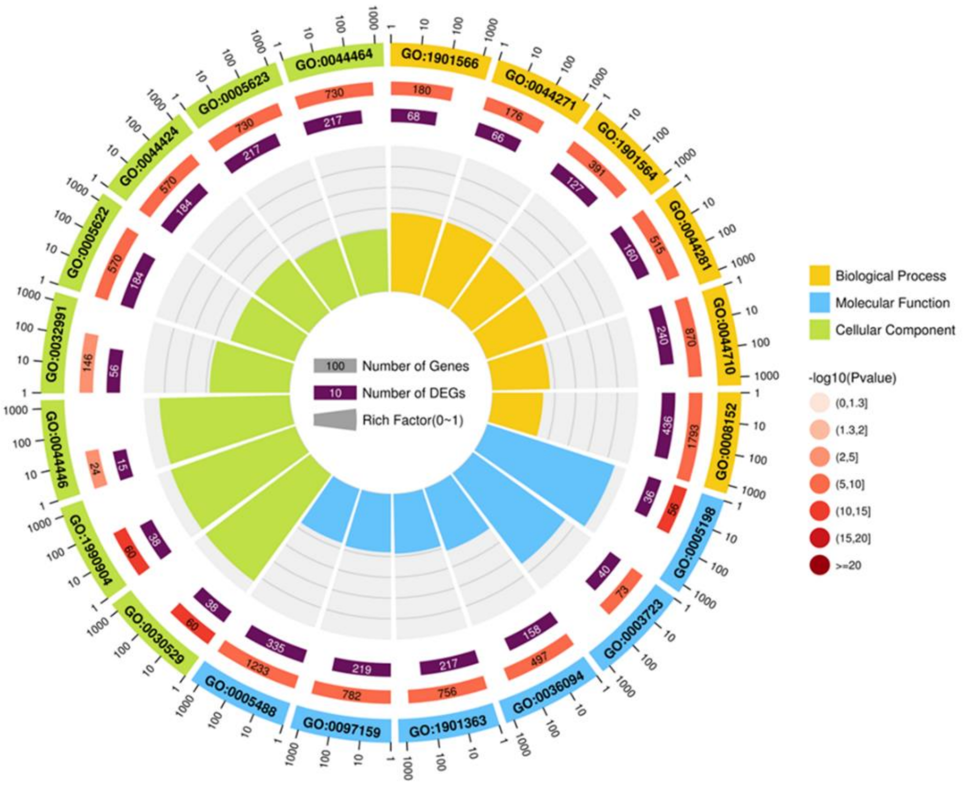

Figure S1
